# Supplementary material for: Workplace Mental Health Status Among Academic Staff: Psychological Distress, Burnout, and Organisational Culture at a South African University
Source: Behav Sci (Basel). 2025 Oct 16;15(10):1410. doi: 10.3390/bs15101410 (PMC12562238; doi:10.3390/bs15101410)
Supplement: Supplementary file 1 [file behavsci-15-01410-s001.zip › behavsci-3833542-supplementary.pdf]

Section B: Institutional Culture regarding mental health

**Please indicate your agreement with the following statements:**						
**1.**	I feel comfortable disclosing information regarding my mental health within the university environment	Strongly disagree	Disagree	Neutral	Agree	Strongly agree
**2.**	SMU provides sufficient training and support to enhance staff mental well-being	Strongly disagree	Disagree	Neutral	Agree	Strongly agree
**3.**	SMU provides adequate mental health services for academic staff	Strongly disagree	Disagree	Neutral	Agree	Strongly agree
**4.**	SMU has established a culture of caring on campus that positively impacts the mental health of academic staff.	Strongly disagree	Disagree	Neutral	Agree	Strongly agree
**5.**	SMU has developed policies and procedures to address mental health concerns among academic staff	Strongly disagree	Disagree	Neutral	Agree	Strongly agree
**6.**	SMU actively promotes equity, diversity, and inclusion in mental health services for academic staff	Strongly disagree	Disagree	Neutral	Agree	Strongly agree
**7.**	SMU empowers academic staff to seek mental health support when needed	Strongly disagree	Disagree	Neutral	Agree	Strongly agree
**8.**	SMU creates positive working practices and conditions for academic staff	Strongly disagree	Disagree	Neutral	Agree	Strongly agree
**9.**	SMU promotes a healthy work/life balance for academic staff	Strongly disagree	Disagree	Neutral	Agree	Strongly agree

10. Do you have any comments to add relating to SMU's institutional culture toward staff mental health?

____

____

____

____

Section C: Needs Assessment for Workplace Health and Wellbeing at SMU

Please indicate your agreement with the following statements					
**1.** I support the implementation of a peer mentorship program at SMU aimed at providing support for mental health and well-being among academic staff	Strongly disagree	Disagree	Neutral	Agree	Strongly agree
**2.** I am interested in accessing online resources or apps that promote mental health and well-being, specifically designed for academic staff	Strongly disagree	Disagree	Neutral	Agree	Strongly agree
**3.** It is important for SMU to foster a culture of open communication and reducing stigma around mental health amongst academic staff	Strongly disagree	Disagree	Neutral	Agree	Strongly agree
**4.** It is important that SMU establish partnerships with external mental health organizations to benefit academic staff	Strongly disagree	Disagree	Neutral	Agree	Strongly agree

5. What types of mental health resources or services would you like to see implemented at SMU (You can choose more than one answer)

- Workshops on stress management and resilience
- Confidential counselling services
- Peer support groups
- Mental health awareness campaigns
- Other (please specify):____

6. What steps can the university take to ensure that mental health support for academic staff is inclusive and addresses the diverse needs of the workforce? (You can choose more than one answer)

- Tailoring support services for different demographics
- Multilingual resources and communication
- Cultural sensitivity training for support providers
- Regular feedback mechanisms for continuous improvement
- Other (please specify):____

7. What organizational changes could enhance the overall mental health and well-being of academic staff within the university? (You can choose more than one answer)

- Flexible working hours
- Telecommuting options (Remote work)
- Clearer communication on expectations
- Clearer communication on workload
- Recognition and acknowledgment of achievements
- Institutional policies promoting work/life balance
- Other (please specify):____

8. Do you have any other suggestions related to academic staff's needs regarding mental health promotion on campus? Please list and/or describe them here:
